# MEK Inhibition Targets Cancer Stem Cells and Impedes Migration of Pancreatic Cancer Cells *In Vitro* and *In Vivo*

**DOI:** 10.1155/2019/8475389

**Published:** 2019-06-02

**Authors:** Karolin Walter, Kanishka Tiwary, Marija Trajkovic-Arsic, Ana Hidalgo-Sastre, Laura Dierichs, Sven T. Liffers, Jiangning Gu, Johann Gout, Lucas-Alexander Schulte, Jan Münch, Thomas Seufferlein, Bruno Sainz, Jens T. Siveke, Eva Rodriguez-Aznar, Patrick C. Hermann

**Affiliations:** ^1^Department of Internal Medicine I, Ulm University, Germany; ^2^Division of Solid Tumor Translational Oncology, West German Cancer Center, University Hospital Essen, Hufelandstrasse 55, 45147 Essen, Germany; ^3^German Cancer Consortium (DKTK, Partner Site Essen) and German Cancer Research Center (DKFZ), Im Neuenheimer Feld 280, 69120 Heidelberg, Germany; ^4^Klinik und Poliklinik für Innere Medizin II, Technical University Munich, Germany; ^5^Institute of Molecular Virology, Ulm University Medical Center, 89081 Ulm, Germany; ^6^Department of Biochemistry, Universidad Autónoma de Madrid (UAM), Madrid, Spain; ^7^Department of Cancer Biology, Instituto de Investigaciones Biomédicas “Alberto Sols” (IIBM), CSIC-UAM, Madrid, Spain; ^8^Chronic Diseases and Cancer, Area 3-Instituto Ramón y Cajal de Investigación Sanitaria (IRYCIS), Madrid, Spain

## Abstract

Pancreatic ductal adenocarcinoma (PDAC) remains a devastating disease with a very poor prognosis. At the same time, its incidence is on the rise, and PDAC is expected to become the second leading cause of cancer-related death by 2030. Despite extensive work on new therapeutic approaches, the median overall survival is only 6-12 months after diagnosis and the 5-year survival is less than 7%. While pancreatic cancer is particularly difficult to treat, patients usually succumb not to the growth of the primary tumor, but to extensive metastasis; therefore, strategies to reduce the migratory and metastatic capacity of pancreatic cancer cells merit close attention. The vast majority of pancreatic cancers harbor RAS mutations. The outstanding relevance of the RAS/MEK/ERK pathway in pancreatic cancer biology has been extensively shown previously. Due to their high dependency on Ras mutations, pancreatic cancers might be particularly sensitive to inhibitors acting downstream of Ras. Herein, we use a genetically engineered mouse model of pancreatic cancer and primary pancreatic cancer cells were derived from this model to demonstrate that small-molecule MEK inhibitors functionally abrogate cancer stem cell populations as demonstrated by reduced sphere and organoid formation capacity. Furthermore, we demonstrate that MEK inhibition suppresses TGF*β*-induced epithelial-to-mesenchymal transition and migration *in vitro* and ultimately results in a highly significant reduction in circulating tumor cells in mice.

## 1. Introduction

Pancreatic ductal adenocarcinoma (PDAC), already one of the deadliest malignancies (currently number 4 in cancer-related deaths), is predicted to become the 2nd most frequent cause of death due to malignancy by 2030 [1]. This exceptional aggressiveness is inextricably linked to the tumor biology of pancreatic cancer and aggravated even more due to (1) late diagnosis as a consequence of the lack of early symptoms, (2) its pronounced resistance to therapy, and (3) its early metastatic spread. The vast majority of patients suffering from pancreatic cancer (up to 80%) are diagnosed at a stage where they are no longer eligible for resection (a potential cure for the disease), making successful chemotherapy an issue of paramount importance and research relevance [2]. However, in spite of extensive efforts to improve therapies, the median survival is still lower than desired, even with the most successful therapies such as FOLFIRINOX (11.1 months) or gemcitabine+nab-paclitaxel (8.5 months) [3, 4].

While resistance to chemotherapy and radiation is one of the hallmarks of pancreatic cancer, early metastatic spread and high metastatic load will eventually kill the patient. We and others have demonstrated the existence of a cancer stem cell (CSC) population in human pancreatic tumors [5, 6], which is ultimately responsible for the propagation and also for the therapy resistance and the metastatic activity of these tumors [5, 7–9].

Metastatic spread is a multifactorial process, involving epithelial-to-mesenchymal transition (EMT), dissociation of tumor cells from the primary tumor, migration, intra- and extravasation, homing, niche formation, and growth at the metastatic site. Recent evidence in the mouse mammary gland suggests that EMT and stemness may be regulated simultaneously by Slug (Snail2), a member of the Snail superfamily of transcription factors [10]. The successful disruption of such signals might therefore result in the simultaneous eradication of CSCs as well as in the abrogation of migrating/metastatic tumor cells. Therefore, in the present study we investigated in detail the effects of MEK inhibitors on EMT and stemness in primary pancreatic cancer (stem) cells.

## 2. Materials and Methods

### 2.1. Mice and Primary Cell Lines

Primary murine pancreatic cancer cell lines were generated as described previously [7]. Briefly, PDAC tumors were resected from Kras^wt/LSL-G12D^;Trp53^loxP/loxP^;Ptf1a^wt/Cre^;LSL-tdRFP^KI/KI^;Slug-YFP (KPCRS) mice expressing an oncogenic Kras mutation [11], a conditional loss of Trp53 [12], an R26-LSL-tdRFP [13] a Cre recombinase under the control of a Ptf1a promoter [14], and a Slug-YFP reporter system [10]. Slug-YFP mice were generously provided by Robert A. Weinberg, Whitehead Institute for Biomedical Research, Cambridge, MA. For the *in vivo* treatment, animals received refametinib (BAY86-9766) as published previously [15]. Primary tumors were minced and digested with collagenase (STEMCELL Technologies, 07902). After fibroblast removal, adherent pancreatic cancer cells were expanded and cultured as previously described [9]. PD0325901 was used at 0.5 *μ*M (5493 cells) or 5 *μ*M (8926 and 9228 cells), and trametinib was used at 0.035 *μ*M (5493) or 0.175 *μ*M (8926 and 9228 cells) unless stated otherwise. TGF*β* was used at 10 nM.

### 2.2. Sphere Formation Assay

Spheres were cultured as described previously [5] in DMEM-F12 (Thermo Fisher Scientific, 10565018) supplemented with B-27 (Thermo Fisher, 17504044) and basic fibroblast growth factor (Novoprotein, CO46). Following three days of PD0325901 treatment, 10,000 cells per milliliter were seeded in ultralow attachment plates (Corning, 3473). After 7 days of incubation, spheres > 40 *μ*m and > 120 *μ*m were quantified using CASY TT (OMNI Life Science, 5651697).

### 2.3. Organoid Cultures

5,000 single cells from mouse primary adherent cell cultures in 25 *μ*l medium were mixed with equal amounts of Matrigel GFR (growth factor reduced, Corning) per well. The culture medium has been described in [16]. Treatment with MEK inhibitors was performed on day 1 or on day 4 for 3 consecutive days. Medium was changed daily. Metabolically active cells were measured with the CellTiter-Glo Luminescent Cell Viability Assay (Promega, G9681) according to the manufacturer's instructions.

### 2.4. Scratch Wound Assays

Cells were grown to confluency and then serum-starved for 24 hours before scratch wounds were made using a sterile 10 *μ*l pipette tip. Subsequently, the cells were cultured with medium containing vehicle or PD0325901 for 24 hours. Images were captured after 24 hours and quantified using ImageJ (version 1.49, https://imagej.nih.gov/ij/).

### 2.5. Migration Assays

Migration assays were performed using inserts with 8 *μ*m pore size PET membranes (Corning, 353097). 5 × 10^4^ cells in serum-free medium were added to the inserts. In the bottom well, media containing 10% FBS were added. After 24 hours, invaded cells were fixed with 4% PFA and stained with DAPI (Merck, 10236276001). Ten random high-power fields were chosen and photographed, and the pictures were quantified using ImageJ.

### 2.6. Flow Cytometry

Flow cytometry analyses were performed using LSR II (BD). Dead cells were excluded using DAPI. Annexin V staining was performed using a BD Annexin V APC kit according to the manufacturer's instructions (BD, 550474). For the identification and quantification of circulating tumor cells in the blood of mice, counting beads (Thermo Fisher Scientific, C36950) were added to the whole blood aspirated from the right ventricle. After red blood cell lysis, samples were stained with an EpCAM-APC antibody (Thermo Fisher) or an appropriate isotype control (BD). The number of cells and beads in the final sample was recorded, and the total quantity of cells in the original sample was calculated. Data were analyzed using FlowJo v10 (Ashland, OR).

### 2.7. Protein Sample Preparation and Western Blotting

Cells were lysed in ice-cold RIPA buffer (Cell Signaling, 9806S) supplemented with PhosSTOp™ (Merck, 4906845001) and a protease inhibitor cocktail (Merck, 11836170001). For each sample, equal amounts of protein were applied to a 12% SDS-polyacrylamide gel and immunoblotted onto PVDF membranes (GE Healthcare, 10600021). Membranes were blocked for 2 hours in 5% BSA in 1x TBST, probed with the indicated primary antibodies (E-cadherin, vimentin, phospho-ERK, ERK, Slug, and Gapdh) overnight at 4°C, washed with 1x TBST, and incubated with a goat anti-rabbit IgG-HRP secondary antibody (Vector Laboratories, BA-1000) for 2 hours. The chemiluminescence detection was performed according to the manufacturer's instructions (Merck, WBKLS0500).

### 2.8. Immunofluorescence

For IF staining, cells were cultured on coverslips (Hecht Assistent 41001115), then fixed with 4% PFA (Sigma), washed with 1x PBST (PBS with 0.3% Triton X-100), and blocked for 1 h at room temperature with blocking solution (10% goat serum and 0.3% Triton X-100 in PBS). An anti-E-cadherin primary antibody (Cell Signaling) was diluted in blocking solution and incubated o/n at 4°C, whereas the secondary antibody (Alexa Fluor 488 goat anti-rabbit, Invitrogen) was diluted in blocking solution and incubated for 2 h at room temperature. All washes were done with 1x PBST. After the final washes, coverslips were mounted with the ProLong Gold antifade reagent with DAPI (Invitrogen) and images were taken using a BioRevo fluorescent microscope (Keyence).

### 2.9. MTT Assay

1,000 cells were seeded in a 96-well plate and incubated for 24 h at 37°C. After 48 h of PD0325901 treatment, cells were incubated for 3 h with 5 mg/ml MTT (Merck, M2128). Finally, DMSO (Roth, A994) was added, and the optical density was measured at 560 nm using an Infinite 200 PRO plate reader (Tecan, Switzerland).

### 2.10. RNAseq

For the RNAseq experiment, primary short-term cultured cell lines generated from PDAC of Ptf1a^wt/Cre^;Kras^wt/LSL-G12D^;Trp53^loxP/loxP^ (CKP) animals [17] were cultivated in standard cell culture dishes and treated with respective IC_50_ concentrations of trametinib (4 cell lines, IC_50_ ranging from 8-25 nM). After 48 h, RNA was isolated using the Maxwell® RSC simplyRNA Cells Kit (Promega) according to the manufacturer's instructions. RNAseq was performed by CeGaT (Tübingen, Germany). Library preparation was performed with the TruSeq Stranded mRNA kit (Illumina), and 2x 100 bp was sequenced on HiSeq 4000 (Illumina). Demultiplexing of the sequencing reads was performed with Illumina CASAVA (2.17). Adapters were trimmed with Skewer (version 0.1.116) (Jiang et al. 2014). RNAseq data were quantified using the quasimapping approach of Salmon [18]. TXImport [19] and DESeq2 [20] were used to import transcript-level counts and to perform differential expression analysis.

### 2.11. RNA Isolation and Real-Time PCR

Total RNA was prepared using the RNeasy kit with on-column genomic DNA digestion following the manufacturer's instructions (Qiagen). First-strand cDNA was prepared using the QuantiTect Reverse Transcription Kit (Qiagen). Reactions were performed with the PerfeCTa SYBR Green FastMix PCR Reagent (Quanta) using a QuantStudio 3 machine (Applied Biosystems). Results were analyzed using the 2^-ddCt^ method relative to *YWHAZ* expression. Reactions were carried out from at least three independent experiments. Primer sequences are provided in the Supplementary Information.

### 2.12. Statistical Analysis

Results for continuous variables are presented as means ± SEM unless stated otherwise. Treatment groups were compared using the Mann-Whitney *U* test unless stated otherwise. *P* values < 0.05 were considered statistically significant. Statistical analyses were performed using GraphPad Prism 5.0 (San Diego, CA).

## 3. Results

### 3.1. MEK Inhibition Compromises the Growth of Murine PDAC Cells

**Figure 1:**
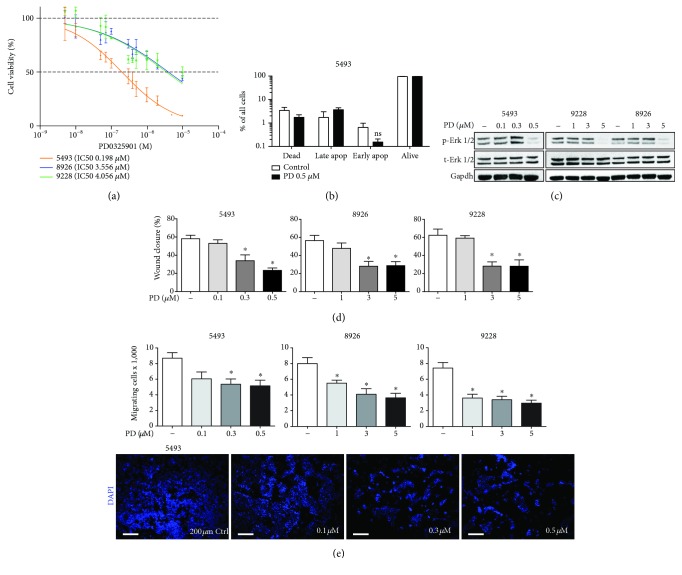
Effect of MEK inhibition on cell viability and migration. (a) MTT assays to determine the respective IC_50_ for three mouse tumor-derived primary cell lines (5493, 8926, and 9228) treated with PD0325901 (PD). The respective IC_50_ is depicted for each cell line. (b) Apoptosis induction under MEK inhibitor treatment (3 days) as measured by annexin V staining and analysis by flow cytometry. (c) Western blot analysis for phosphorylated and total ERK 1/2 was performed on the three cell lines treated with PD0325901 at the indicated concentrations. Gapdh was used as a loading control. (d) The percentage of wound closure 24 h after scratch wound induction in primary cells treated with vehicle or PD0325901 at the indicated concentrations. (e) Quantification and representative micrographs (10x, DAPI nuclear staining) of Transwell migration assays with vehicle or PD0325901 treatment with the indicated concentrations. *n* ≥ 3 for all experiments, *n* = 2 for Western blot. ^∗^ *P* < 0.05 vs. control. ns = not significant.

We first evaluated the effects of the small-molecule MEK inhibitor PD0325901 on primary cell lines derived from KPCRS mice. MTT assays revealed a dose-dependent response to MEK inhibition on the utilized primary cells, demonstrating their dependency on a functional RAS-RAF-MEK-ERK pathway. Interestingly, the different cells displayed a variable responsiveness to PD0325901 (Figure 1(a)). Further experiments with each cell line were performed using PD0325901 concentrations slightly above the respective IC_50_. We were able to demonstrate next that at the utilized concentrations, no significant changes in apoptosis or cell death were detected in two of these cell lines after 72 h of treatment (Figure 1(b), Supp. Fig.  1A). Furthermore, we found phosphorylation of ERK, as a downstream target to MEK, to be abrogated upon treatment with MEK inhibitors, confirming the effectiveness of the compound in our model system (Figure 1(c)).

### 3.2. MEK Inhibition Decreases Migration in a Dose-Dependent Manner

Pancreatic cancer is characterized by early metastatic spread through cells with increased migratory properties. In order to delineate the role of MEK signaling in cell migration, we performed scratch wound assays on three primary tumor cell lines. MEK inhibition resulted in significantly reduced “wound closure” (i.e., migration capacity) in all primary cell lines. The reduction in migratory activity was clearly dose-dependent (Figure 1(d), representative pictures of two cell lines in Suppl. Fig.  1B). Since scratch wound assays are error-prone due to proliferation effects, we used more reliable Transwell migration assays to further investigate the effect of MEK inhibition on cell migration. After pretreatment with PD0325901, a significant reduction in migration was observed (Figure 1(e)). These results indicate that MEK signaling is essential for the migratory activity in PDAC cells.

### 3.3. MEK Inhibition Ablates TGF*β*-Induced EMT

**Figure 2:**
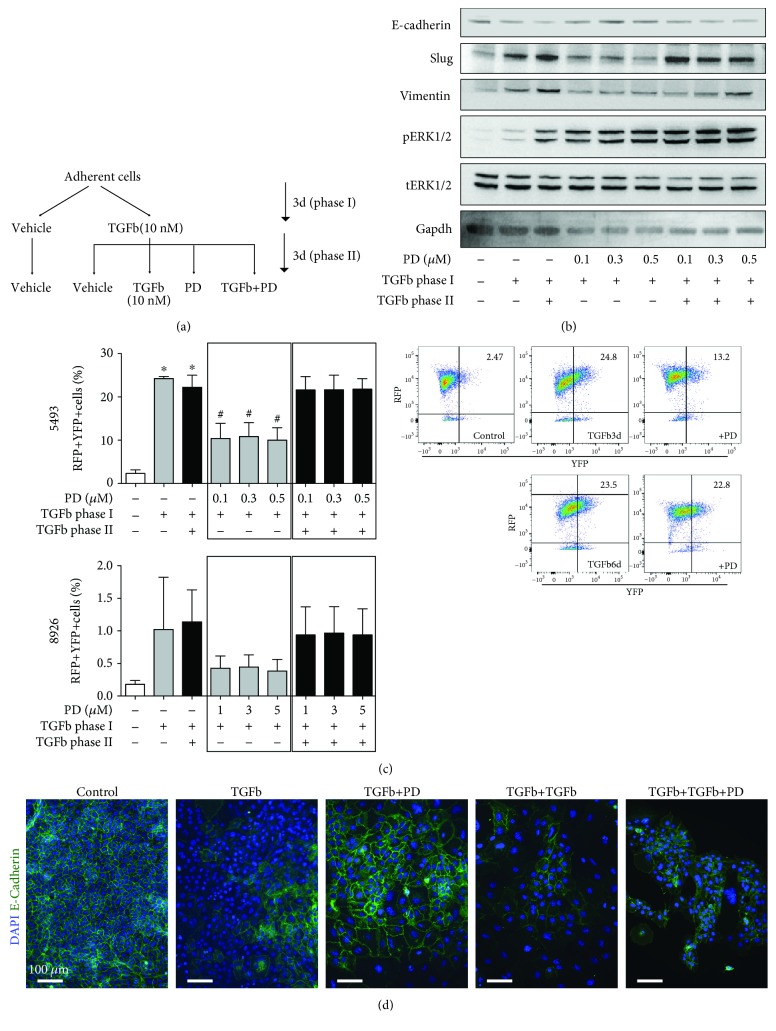
Effects of MEK inhibition on TGF*β*-induced EMT. (a) Experimental overview for TGF*β* and PD0325901 co–treatment. (b) Western blot analysis of key proteins involved in EMT with treatment *in vitro*. Gapdh was used as a loading control. (c) Percentage of RFP+YFP+ cells under treatment as indicated and representative cytometry blots. (d) Immunofluorescence micrographs of E-cadherin expression with treatment as indicated. *n* ≥ 3 for all experiments, *n* ≥ 2 for Western blots. ^∗^ *P* < 0.05 vs. control, ^#^ *P* < 0.05 vs. TGF*β*.

Transforming growth factor beta (TGF*β*) promotes tumor progression in advanced cancer stages by inducing tumor growth, but most importantly inducing metastasis through activation of EMT, resulting in increased invasion and metastasis [21] via upregulation of transcription factors such as the zinc finger proteins Snail and Slug [22]. The cell lines we used in this study are primary tumor cells derived from a mouse model which spontaneously develops metastatic PDAC and reports pancreatic and pancreas-derived cells by Ptf1a-mediated RFP expression and reports Slug activity via YFP expression. Given the significant role of TGF*β* in EMT induction and subsequent metastasis, we wondered whether MEK inhibition could abrogate an active EMT program, initiated by TGF*β*. We therefore treated the cells with TGF*β* for 3 or 6 days (experimental overview in Figure 2(a)). PD0325901 was added after 3 days of pretreatment.

We then measured treatment effects by Western blotting of Slug and vimentin, which were strongly upregulated following TGF*β* treatment. Indeed, subsequent MEK inhibition greatly reduced Slug protein levels (Figure 2(b)). Interestingly, MEK inhibition was unable to overcome the effects of continuous TGF*β* stimulation. Furthermore, we made use of the Slug-YFP reporter system in our cells: TGF*β* treatment resulted in a significant induction of EMT as evidenced by robust Slug-YFP expression, i.e., a high increase in RFP^+^YFP^+^ cells after 3 days and 6 days (Figure 2(c)). Interestingly, MEK inhibition with PD0325901 significantly diminished this RFP^+^YFP^+^ population by almost 50% after 3 days of TGF*β* treatment; however, by matching the observation in Western blotting, MEK inhibition was not able to abrogate the effects of continuous TGF*β* treatment.

In line with the previous experiments, immunofluorescence staining for E-cadherin revealed that while TGF*β* treatment suppressed E-cadherin, treatment with PD0325901 resulted in reexpression of E-cadherin, indicating induction of a more epithelial cell phenotype (Figure 2(d)).

Altogether, the above *in vitro* results indicate that pharmacological inhibition of MEK inhibits TGF*β*-induced EMT and migration *in vitro*.

### 3.4. MEK Inhibitors Target Pancreatic Cancer Stem Cells

**Figure 3:**
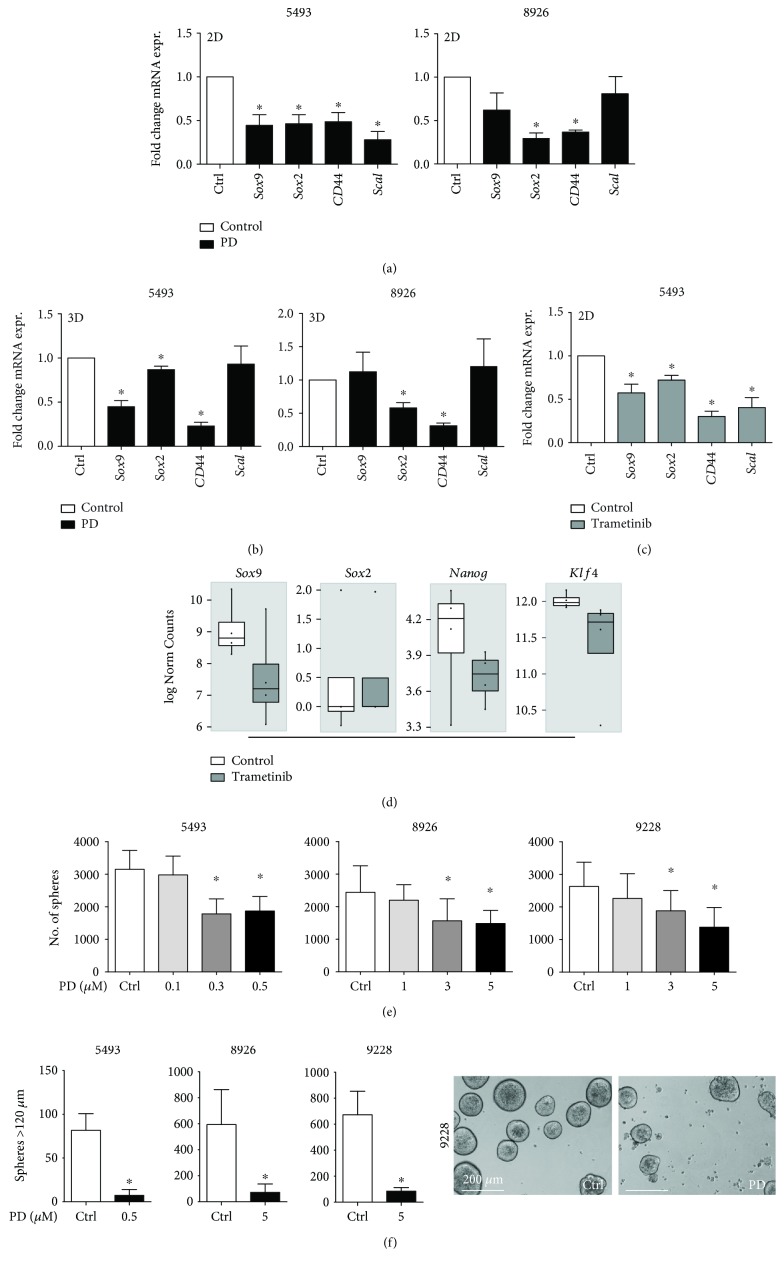
Effects of MEK inhibition on stemness. Gene expression of the indicated stemness-associated genes in (a) adherent cultures or (b) spheres after treatment with vehicle or PD0325901 (PD). (c) Gene expression and (d) RNAseq analysis of murine primary cancer cell lines treated with DMSO vehicle (white) or trametinib (grey) for 48 h. (e) Sphere formation capacity of cells pretreated with PD0325901 at the indicated concentrations. (f) Quantification of large spheres (>120 *μ*m) and representative micrographs of sphere cultures after 7 days. *n* ≥ 3 for all experiments. ^∗^ *P* < 0.05 vs. control.

Increased migration and invasion are key features promoted by EMT, which, in turn, have been shown to also confer stemness properties [10]. Therefore, we investigated the expression of genes associated with pluripotency and stemness upon treatment. Indeed, we observed a significant downregulation in *Sox9*, *Sox2*, *CD44*, and *Sca1* in adherent cell cultures (Figure 3(a)). Sphere cultures are enriched for cancer stem cells, i.e., tumor cells with stem cell-like features, which show unlimited self-renewal and are resistant to chemotherapeutics [5]. Even in 3D sphere cultures, we observed comparable, albeit slightly less pronounced effects of PD0325901 on stemness-associated genes as in monolayer cultures (Figure 3(b)). Treatment with the clinically relevant MEK inhibitor trametinib also resulted in significant downregulation of stemness-associated genes (Figure 3(c)). In order to generalize our approach to more primary cell lines, we performed RNAseq on 4 additional trametinib- vs. vehicle-treated KPC-derived primary mouse cell lines. The subsequent analysis revealed downregulation of *Nanog*, *Sox9*, and *Klf4* (Figure 3(d)), matching the qRT-PCR dataset.

In order to elucidate the functional effects of MEK inhibition on CSCs, we performed sphere formation assays after 72 h of pretreatment with PD0325901. The number of spheres formed was significantly reduced after MEK inhibition, and the size of the spheres formed after treatment was found to be notably smaller compared to the vehicle-treated control (Figures 3(e) and 3(f)).

### 3.5. MEK Inhibitors Prevent Organoid Formation and Decrease CTCs *In Vivo*

**Figure 4:**
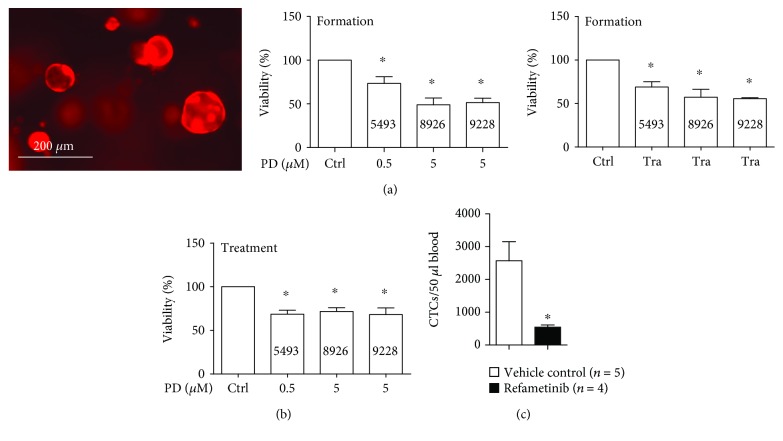
MEK inhibitors diminish organoid formation and eliminate CTCs *in vivo.* (a) Representative micrograph of RFP+ organoid cultures (left, 5493 cells). Organoid formation under treatment with PD0325901 (PD) (middle) and trametinib (Tra) (right) in 3 primary cell lines treated as indicated. (b) Viability of preformed organoids with PD0325901 treatment *in vitro*. (c) Absolute numbers of circulating tumor cells (CTCs) in CKP mice treated with either vehicle control or refametinib as described in [15]. *n* ≥ 3 for all experiments. ^∗^ *P* < 0.05 vs. control.

3D organoid cultures are a more physiological cell culture model than 2D monolayer cultures and better reflect *in vivo* conditions by maintaining cell-to-cell signaling. Thus, organoids better recapitulate the original tumor and are preferable to predict treatment response as compared to monolayer cultures [23]. Therefore, we generated 3D organoid cultures from our primary cell lines and performed organoid formation and treatment experiments *in vitro* (Figure 4(a)). As expected, we observed a significantly decreased organoid formation with MEK inhibition treatment. This holds true for the formation of organoids under treatment with PD0325901 or trametinib (Figure 4(a)), as well as for the treatment of already established organoids (Figure 4(b)).

As a functional *in vivo* readout for efficacy of MEK inhibitors on PDAC cell migration, we quantified CTCs in KPC mice treated with another clinical-grade MEK inhibitor, refametinib. Refametinib is a potent MEK1/2 inhibitor with beneficial effects in the treatment of pancreatic cancer patients [24]. For this purpose, we extracted blood from the right ventricle of CKP mice treated either with refametinib or with vehicle control (the detailed experimental setup, tumor growth data, and imaging of the primary tumor have already been published in [15]). In agreement with the *in vitro* data, we observed significantly fewer CTCs in the blood stream of these mice after refametinib treatment (Figure 4(c)).

## 4. Discussion

Using primary cancer cells derived from genetically engineered mice that spontaneously develop PDAC, we investigated the effects of MEK inhibition on stemness, migration, and circulating tumor cells. PD0325901-mediated MEK inhibition *in vitro* compromised the growth and survival of the cells. This is not surprising, as MEK inhibition has already been described to induce the intrinsic apoptotic pathway in different contexts [25–28]. However, in our study, the cells are viable after treatment and showed no significant differences with regard to apoptosis. To exclude a bias due to a proliferation disadvantage, we performed subsequent migration and sphere formation experiments with matching cell numbers after pretreatment, thus ensuring an equal starting point regarding the number of cells in each condition.

MEK inhibition *in vitro* impaired the invasion and migration capacities of PDAC cells. Mechanistically, we show that these effects are mediated via inhibition of TGF*β*-induced EMT, which plays a crucial role during development, and is upregulated in pathological conditions such as fibrosis and tumor progression in adults (reviewed in [29]). EMT also regulates many downstream molecules that are critical for cell survival, cell cycle progression, and epithelial integrity [30–32]. Specifically, loss of the epithelial cell-cell adhesion molecule E-cadherin is considered a hallmark of EMT, potentiating invasion and metastasis (reviewed in [33]).

The MAPK pathway has been shown to drive the expression of EMT-related transcription factors, in particular that of the Snail superfamily members during development [34, 35], fibrosis [36], and cancer progression and migration [37]. Additionally, it cooperates with other proteins of the TGF*β* family, which can initiate and maintain EMT in different contexts (reviewed in [38]). Importantly, TGF*β* upregulation has frequently been reported in human carcinomas (reviewed in [21]); however, a clear relationship between MEK activity and a migratory and invasive phenotype in PDAC has not been described thus far. Here, we show that the MAPK signaling pathway, acting through MEK, confers invasive properties to the cells by regulating the transcription factor Slug. Interestingly, however, MEK inhibition could not overcome the effects of sustained TGF*β* activation on EMT, indicating that these two pathways, although capable of cooperating, act through different downstream effectors or that compensatory feedback loop mechanisms play a relevant role.

Importantly, the activation of MAPK signaling components can confer stemness properties to cells [7, 39]. Recent advances in understanding PDAC progression have led to the identification of CSCs by us [5] and others [6]. These cells represent a subpopulation of cancer cells with features typically associated with stem cells, such as unlimited self-renewal. These cells are also responsible for tumor progression and therapy resistance, but most importantly, via a population of migrating CSCs, they are indispensable for metastatic spread (reviewed in [40]). For human pancreatic cancers, different markers or marker sets have been proposed for the identification of CSCs [5, 6, 41]. Similarly, no unified marker set to identify CSCs in mice has been published to date, while several candidate surface markers (or combinations thereof) have been proposed [42–44]. Therefore, CSCs need to be identified operationally, making sphere formation and colony formation assays valuable tools for the identification of CSCs. Here, we show that MEK inhibition functionally inhibits CSC populations as evidenced by significantly reduced sphere formation capacity, a surrogate marker for CSC activity. Since we do not observe unspecific cytotoxicity, the data in this study indicate that CSCs are more MEK-dependent than the general cell population. This is further corroborated by the observation that stemness- and pluripotency-associated markers are significantly downregulated upon MEK inhibitor treatment.

The investigation of treatment effects on primary organoid cultures is a very promising way to determine treatment efficacy in a more physiological setting. Here, 3D clusters of cells rather than monolayers were treated, giving credit to cell-cell interactions and paracrine signaling during treatment. While this method has been shown to predict treatment response [23, 45], its validity for determining effects on CSC populations has not been conclusively demonstrated. We here demonstrate that the capacity of primary PDAC tumor cells to form spheres and organoids is compromised under MEK inhibition, suggesting a decrease in their tumor-initiating potential. We also obtained similar results when the treatment was performed in already formed organoids, further validating our *in vitro* data and the usefulness of this setup for drug testing.

Activating mutations in Kras promote proliferation and survival through the RAF/MEK/ERK and PIK3/AKT pathways. Kras^G12D^ mutations, as investigated in our study, are the most prevalent mutations in pancreatic cancer [46], but due to the inherent nature of the Ras protein, Ras inhibition has not resulted in relevant clinical benefit despite high prevalence of Ras mutations in pancreatic cancer. Therefore, therapies designed to specifically target downstream effectors have been developed (reviewed in [47]). MEK inhibitors have already received much attention, as they are able to decrease tumor formation in animal models, particularly in pancreatic cancer [15]. Here, we show for the first time that MEK inhibition also significantly reduces the number of CSCs, organoids, and circulating tumor cells (CTCs) *in vivo*. While CTC numbers do not necessarily correlate directly with the metastatic load in a patient, they are a strong indicator of prognosis [48]. Since primary tumors significantly decreased in size with refametinib treatment (previously published in [15]), the observed effects on CTC numbers could (at least partially) also be due to a general reduction in tumor size. However, the significant reduction of Slug-expressing cells *in vitro* suggests a strong inhibition of TGF*β*-induced EMT with MEK inhibition, which in turn would result in the abrogation of CTCs. This offers a possible mechanism by which MEK can exert its function promoting survival, migration, invasion, stemness, and CTC initiation, contributing to the relevance of MEK in PDAC and of MEK inhibitors as therapeutic options.

To date, clinical trials using several MEK inhibitors have shown poor bioavailability, high toxicity, and/or low antitumor activity, likely due to the rapid development of resistance. Ongoing clinical trials with newly developed MEK inhibitors alone or in combination with other treatments have proven to be more efficient (reviewed in [49, 50]). Therefore, MEK inhibition together with that of other relevant pathways, such as TGF*β*, may still be promising for treating PDAC, especially in combination with chemotherapy, and should be further evaluated.

## Data Availability

The data used to support the findings of this study are available from the corresponding author upon reasonable request.

## Supplementary Materials

Supplementary Materials Suppl. Information: primer sequences and utilized antibodies. Supplementary Figure 1: (A) apoptosis induction in 8926 cells with MEK inhibitor treatment as measured by annexin V staining and analysis by flow cytometry. (B) Representative micrographs for 5493 and 8926 cells of scratch wounds at the start of the experiment (0 h) and after 24 h when measured for analysis.

## References

[1] Rahib L., Smith B. D., Aizenberg R., Rosenzweig A. B., Fleshman J. M., Matrisian L. M. (2014). Projecting cancer incidence and deaths to 2030: the unexpected burden of thyroid, liver, and pancreas cancers in the United States. *Cancer Research*.

[2] Brand R. E., Tempero M. A. (1998). Pancreatic cancer. *Current Opinion in Oncology*.

[3] Conroy T., Desseigne F., Ychou M. (2011). FOLFIRINOX versus gemcitabine for metastatic pancreatic cancer. *The New England Journal of Medicine*.

[4] Von Hoff D. D., Ervin T., Arena F. P. (2013). Increased survival in pancreatic cancer with nab-paclitaxel plus gemcitabine. *The New England Journal of Medicine*.

[5] Hermann P. C., Huber S. L., Herrler T. (2007). Distinct populations of cancer stem cells determine tumor growth and metastatic activity in human pancreatic cancer. *Cell Stem Cell*.

[6] Li C., Heidt D. G., Dalerba P. (2007). Identification of pancreatic cancer stem cells. *Cancer Research*.

[7] Hermann P. C., Sancho P., Cañamero M. (2014). Nicotine promotes initiation and progression of KRAS-induced pancreatic cancer via *Gata6*-dependent dedifferentiation of acinar cells in mice. *Gastroenterology*.

[8] Hermann P. C., Trabulo S. M., Sainz B. (2013). Multimodal treatment eliminates cancer stem cells and leads to long-term survival in primary human pancreatic cancer tissue xenografts. *PLoS One*.

[9] Lonardo E., Hermann P. C., Mueller M.-T. (2011). Nodal/activin signaling drives self-renewal and tumorigenicity of pancreatic cancer stem cells and provides a target for combined drug therapy. *Cell Stem Cell*.

[10] Guo W., Keckesova Z., Donaher J. L. (2012). Slug and Sox9 cooperatively determine the mammary stem cell state. *Cell*.

[11] Jackson E. L., Willis N., Mercer K. (2001). Analysis of lung tumor initiation and progression using conditional expression of oncogenic K-ras. *Genes & Development*.

[12] Marino S., Vooijs M., van der Gulden H., Jonkers J., Berns A. (2000). Induction of medulloblastomas in *p53*-null mutant mice by somatic inactivation of Rb in the external granular layer cells of the cerebellum. *Genes & Development*.

[13] Luche H., Weber O., Nageswara Rao T., Blum C., Fehling H. . J. (2007). Faithful activation of an extra-bright red fluorescent protein in "knock-in" Cre-reporter mice ideally suited for lineage tracing studies. *European Journal of Immunology*.

[14] Kawaguchi Y., Cooper B., Gannon M., Ray M., MacDonald R. J., Wright C. V. E. (2002). The role of the transcriptional regulator Ptf1a in converting intestinal to pancreatic progenitors. *Nature Genetics*.

[15] Trajkovic-Arsic M., Heid I., Steiger K. (2017). Apparent diffusion coefficient (ADC) predicts therapy response in pancreatic ductal adenocarcinoma. *Scientific Reports*.

[16] Reichert M., Takano S., Heeg S., Bakir B., Botta G. P., Rustgi A. K. (2013). Isolation, culture and genetic manipulation of mouse pancreatic ductal cells. *Nature Protocols*.

[17] Mazur P. K., Herner A., Mello S. S. (2015). Combined inhibition of BET family proteins and histone deacetylases as a potential epigenetics-based therapy for pancreatic ductal adenocarcinoma. *Nature Medicine*.

[18] Patro R., Duggal G., Love M. I., Irizarry R. A., Kingsford C. (2017). Salmon provides fast and bias-aware quantification of transcript expression. *Nature Methods*.

[19] Soneson C., Love M. I., Robinson M. D. (2015). Differential analyses for RNA-seq: transcript-level estimates improve gene-level inferences. *F1000Res*.

[20] Love M. I., Huber W., Anders S. (2014). Moderated estimation of fold change and dispersion for RNA-seq data with DESeq2. *Genome Biology*.

[21] Massague J. (2008). TGF*β* in cancer. *Cell*.

[22] Brandl M., Seidler B., Haller F. (2010). IKK*α* controls canonical TGF*β*–SMAD signaling to regulate genes expressing SNAIL and SLUG during EMT in panc1 cells. *Journal of Cell Science*.

[23] Boj S. F., Hwang C. I., Baker L. A. (2015). Organoid models of human and mouse ductal pancreatic cancer. *Cell*.

[24] Van Laethem J.-L., Riess H., Jassem J. (2017). Phase I/II study of refametinib (BAY 86-9766) in combination with gemcitabine in advanced pancreatic cancer. *Targeted Oncology*.

[25] Chen D., Wei L., Yu J., Zhang L. (2014). Regorafenib inhibits colorectal tumor growth through PUMA-mediated apoptosis. *Clinical Cancer Research*.

[26] de la Puente P., Muz B., Jin A. (2016). MEK inhibitor, TAK-733 reduces proliferation, affects cell cycle and apoptosis, and synergizes with other targeted therapies in multiple myeloma. *Blood Cancer Journal*.

[27] Hoshino R., Tanimura S., Watanabe K., Kataoka T., Kohno M. (2001). Blockade of the extracellular signal-regulated kinase pathway induces marked G1 cell cycle arrest and apoptosis in tumor cells in which the pathway is constitutively activated: up-regulation of p27(Kip1). *The Journal of Biological Chemistry*.

[28] Meng J., Dai B., Fang B. (2010). Combination treatment with MEK and AKT inhibitors is more effective than each drug alone in human non-small cell lung cancer in vitro and in vivo. *PLoS One*.

[29] Thiery J. P., Acloque H., Huang R. Y. J., Nieto M. A. (2009). Epithelial-mesenchymal transitions in development and disease. *Cell*.

[30] Inukai T., Inoue A., Kurosawa H. (1999). SLUG, a ces-1-related zinc finger transcription factor gene with antiapoptotic activity, is a downstream target of the E2A-HLF oncoprotein. *Molecular Cell*.

[31] Kajita M., McClinic K. N., Wade P. A. (2004). Aberrant expression of the transcription factors snail and slug alters the response to genotoxic stress. *Molecular and Cellular Biology*.

[32] Vega S., Morales A. V., Ocaña O. H., Valdés F., Fabregat I., Nieto M. A. (2004). Snail blocks the cell cycle and confers resistance to cell death. *Genes & Development*.

[33] Hanahan D., Weinberg R. A. (2011). Hallmarks of cancer: the next generation. *Cell*.

[34] Ciruna B., Rossant J. (2001). FGF signaling regulates mesoderm cell fate specification and morphogenetic movement at the primitive streak. *Developmental Cell*.

[35] Geary L., LaBonne C. (2018). FGF mediated MAPK and PI3K/Akt signals make distinct contributions to pluripotency and the establishment of neural crest. *eLife*.

[36] Strippoli R., Loureiro J., Moreno V. (2015). Caveolin-1 deficiency induces a MEK-ERK1/2-Snail-1-dependent epithelial-mesenchymal transition and fibrosis during peritoneal dialysis. *EMBO Molecular Medicine*.

[37] Lemieux E., Bergeron S., Durand V., Asselin C., Saucier C., Rivard N. (2009). Constitutively active MEK1 is sufficient to induce epithelial-to-mesenchymal transition in intestinal epithelial cells and to promote tumor invasion and metastasis. *International Journal of Cancer*.

[38] Zavadil J., Bottinger E. P. (2005). TGF-beta and epithelial-to-mesenchymal transitions. *Oncogene*.

[39] Cheng J., Liu C., Liu L. (2016). MEK1 signaling promotes self-renewal and tumorigenicity of liver cancer stem cells via maintaining SIRT1 protein stabilization. *Oncotarget*.

[40] Valle S., Martin-Hijano L., Alcalá S., Alonso-Nocelo M., Sainz Jr B. (2018). The ever-evolving concept of the cancer stem cell in pancreatic cancer. *Cancers*.

[41] Miranda-Lorenzo I., Dorado J., Lonardo E. (2014). Intracellular autofluorescence: a biomarker for epithelial cancer stem cells. *Nature Methods*.

[42] Rovira M., Scott S. G., Liss A. S., Jensen J., Thayer S. P., Leach S. D. (2010). Isolation and characterization of centroacinar/terminal ductal progenitor cells in adult mouse pancreas. *Proceedings of the National Academy of Sciences of the United States of America*.

[43] Lynn F. C., Smith S. B., Wilson M. E., Yang K. Y., Nekrep N., German M. S. (2007). Sox9 coordinates a transcriptional network in pancreatic progenitor cells. *Proceedings of the National Academy of Sciences of the United States of America*.

[44] Bailey J. M., Alsina J., Rasheed Z. A. (2014). DCLK1 marks a morphologically distinct subpopulation of cells with stem cell properties in preinvasive pancreatic cancer. *Gastroenterology*.

[45] Fujii M., Clevers H., Sato T. (2019). Modeling human digestive diseases with CRISPR-Cas9-modified organoids. *Gastroenterology*.

[46] Brauswetter D., Gurbi B., Varga A. (2017). Molecular subtype specific efficacy of MEK inhibitors in pancreatic cancers. *PLoS One*.

[47] Rajalingam K., Schreck R., Rapp U. R., Albert Š. (2007). Ras oncogenes and their downstream targets. *Biochimica et Biophysica Acta (BBA) - Molecular Cell Research*.

[48] Cristofanilli M., Budd G. T., Ellis M. J. (2004). Circulating tumor cells, disease progression, and survival in metastatic breast cancer. *The New England Journal of Medicine*.

[49] Luke J. J., Ott P. A., Shapiro G. I. (2014). The biology and clinical development of MEK inhibitors for cancer. *Drugs*.

[50] Neuzillet C., Tijeras-Raballand A., de Mestier L., Cros J., Faivre S., Raymond E. (2014). MEK in cancer and cancer therapy. *Pharmacology & Therapeutics*.

