# Supplementary material for: MEK Inhibition Targets Cancer Stem Cells and Impedes Migration of Pancreatic Cancer Cells In Vitro and In Vivo
Source: Stem Cells Int. 2019 Jun 2;2019:8475389. doi: 10.1155/2019/8475389 (PMC6589314; doi:10.1155/2019/8475389)

Suppl. Information

A

Primer sequences:

**Snai2**	F: TCTGCAGACCCACTCTGATG, R: AGCAGCCAGACTCCTCATGT
**Sox9**	F: AGGAAGCTGGCAGACCAGTA, R: TCCACGAAGGGTCTCTTCTC
**Sox2**	QT00249347, Qiagen
**Sca1**	F: CCATCAATTACCTGCCCCTA, R: GGCAGATGGGTAAGCAAAGA
**CD44**	F: GTGGGCAGAAGAAAAAGCTG R: TTGTTCACCAAATGCACCAT
**E-Cadherin**	F: GCTGGCTGAAAGTGACACAG R: TATCTCCATGGGATCCTCCA
**Vimentin**	F: TGAAGGAAGAGATGGCTCGT R: TCCAGCAGCTTCCTGTAGGT
**YWHAZ (HK)**	F; TGCTGAGAAAAAGCAGCAGA , R: GAAGCATTGGGGATCAAGAA

B

Antibodies:

No.	Primary Antibody	Concentration	Secondary Antibody	Cat. Number	Company
1	E-cadherin	1:1000 (1:100 for IF)	anti rabbit	24E10	Cell Signaling
2	Vimentin	1:1000	anti rabbit	D21H3	Cell Signaling
3	p44/42 MAPK (ERK 1/2)	1:1000	anti rabbit	1375F5	Cell Signaling
4	phospho-p44/42 MAPK (T202/Y204)	1:1000	anti rabbit	D13.14.4E	Cell Signaling
5	Slug	1:000	anti rabbit	C19G7	Sigma
6	GAPDH	1:10,000	Anti-Rabbit	G9545	Cell Signaling
7	GAPDH	1:10,000	Anti-Mouse	C65-5G4	HyTest Ltd.
8	Goat anti-rabbit IgG	1:2000		G21234	Life Technologies
9	ECL™ anti-mouse HRP	1:2000		NA931V	GE Healthcare

* $P < 0.05$ vs Ctrl

Suppl. Figure 1

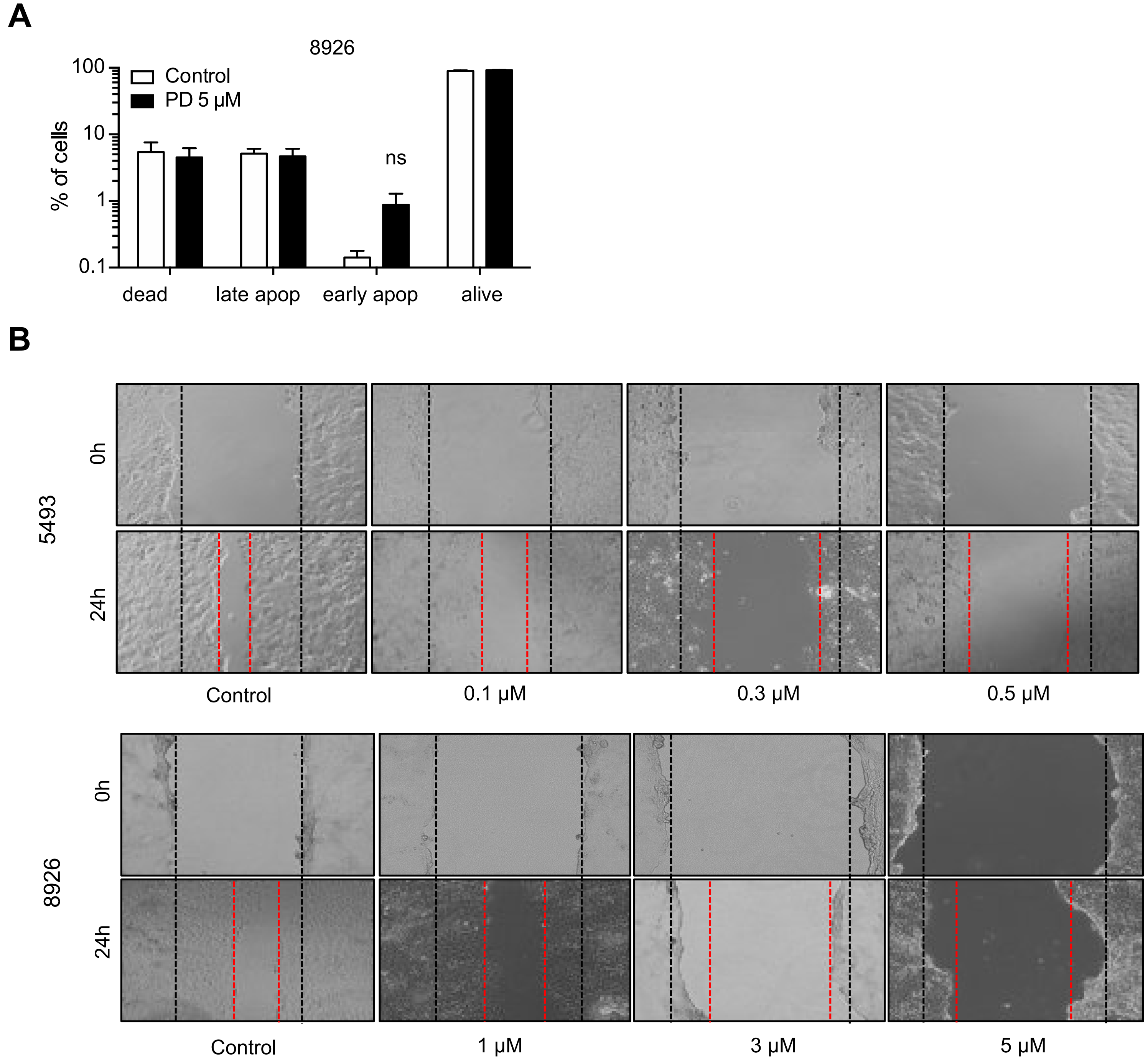

Supplement: Supplementary Materials — Suppl. Information: primer sequences and utilized antibodies. Supplementary Figure 1: (A) apoptosis induction in 8926 cells with MEK inhibitor treatment as measured by annexin V staining and analysis by flow cytometry. (B) Representative micrographs for 5493 and 8926 cells of scratch wounds at the start of the experiment (0 h) and after 24 h when measured for analysis. [file 8475389.f1.pdf]
